# Health economic analysis of polygenic risk score use in primary prevention of coronary artery disease – A system dynamics model

**DOI:** 10.1016/j.ajpc.2024.100672

**Published:** 2024-05-18

**Authors:** Stephen T. Vernon, Stuart Brentnall, Danielle J Currie, Cindy Peng, Michael P. Gray, Giordano Botta, Deo Mujwara, Stephen J. Nicholls, Stuart M. Grieve, Julie Redfern, Clara Chow, Jean-Frederic Levesque, Peter J. Meikle, Garry Jennings, Zanfina Ademi, Andrew Wilson, Gemma A. Figtree

**Affiliations:** a Cardiovascular Discovery Group, Kolling Institute of Medical Research, University of Sydney, Australia; b Department of Cardiology, Royal North Shore Hospital, Australia; c Northern Clinical School, Faculty of Medicine and Health, University of Sydney, Australia; d Decision Analytics, The SAX Institute, Sydney, Australia; e Allelica Inc, New York, United States; f Monash Cardiovascular Research Centre, Monash University, Melbourne, Victoria, Australia; g Imaging and Phenotyping Laboratory, Charles Perkins Centre, University of Sydney, Sydney, NSW, Australia; h Sydney Medical School and School of Health Sciences, Faculty of Medicine and Health, University of Sydney, Sydney, NSW, Australia; i School of Health Sciences, Faculty of Medicine and Health, University of Sydney, Sydney, New South Wales, Australia; j Westmead Applied Research Centre (C.K.C.), University of Sydney, Australia; k NSW Health, Sydney, NSW, Australia; l Centre for Primary Health Care and Equity, University of New South Wales, Sydney, NSW, Australia; m Baker Heart and Diabetes Institute, Melbourne, VIC, Australia; n Monash University, Melbourne, VIC, 3800, Australia; o University of Sydney, Sydney, NSW, Australia; p Centre for Medicine Use and Safety, Faculty of Pharmacy and Pharmaceutical Sciences, Monash University, Melbourne, Australia; q School of Public Health and Preventive Medicine, Monash University, Melbourne, Australia; r Menzies Centre for Health Policy and Economics, Faculty of Medicine and Health, School of Public Health, The University of Sydney, Sydney, Australia

**Keywords:** Health economics, Polygenic risk score, Coronary artery disease, Cardiovascular disease

## Abstract

**Background:**

Primary prevention programs utilising traditional risk scores fail to identify all individuals who suffer acute cardiovascular events. We aimed to model the impact and cost effectiveness of incorporating a Polygenic risk scores (PRS) into the cardiovascular disease CVD primary prevention program in Australia, using a whole-of-system model.

**Methods:**

System dynamics models, encompassing acute and chronic CVD care in the Australian healthcare setting, assessing the cost-effectiveness of incorporating a CAD-PRS in the primary prevention setting. The time horizon was 10-years.

**Results:**

Pragmatically incorporating a CAD-PRS in the Australian primary prevention setting in middle-aged individuals already attending a Heart Health Check (HHC) who are determined to be at low or moderate risk based on the 5-year Framingham risk score (FRS), with conservative assumptions regarding uptake of PRS, could have prevented 2, 052 deaths over 10-years, and resulted in 24, 085 QALYs gained at a cost of $19, 945 per QALY with a net benefit of $724 million. If all Australians overs the age of 35 years old had their FRS and PRS performed, and acted upon, 12, 374 deaths and 60, 284 acute coronary events would be prevented, with 183, 682 QALYs gained at a cost of $18, 531 per QALY, with a net benefit of $5, 780 million.

**Conclusions:**

Incorporating a CAD-PRS in a contemporary primary prevention setting in Australia would result in substantial health and societal benefits and is cost-effective. The broader the uptake of CAD-PRS in the primary prevention setting in middle-aged Australians, the greater the impact and the more cost-effective the strategy.

## 1 Introduction

Cardiovascular disease (CVD) primary prevention programs that utilise multivariable risk models incorporating the standard modifiable cardiovascular risk factors (SMuRFs)—including smoking, hypertension, diabetes mellitus, and hypercholesterolaemia—in order to identify individuals at risk of CVD who will benefit from lifestyle and pharmacotherapy interventions, have been developed, implemented and refined over recent decades. Such programs have led to substantial decreases in CVD morbidity and mortality; yet coronary heart disease remains the leading cause of death worldwide [1]. Despite the success of these programs at a community- and population-level, an increasing body of literature is recognising the importance of people with coronary artery disease (CAD) without such SMuRFs. Recent studies have shown that up to 25 % of patients who present with ST elevation myocardial infarction (STEMI) having developed atherosclerosis despite no traditional risk factors, and are therefore currently unable to benefit from standard primary prevention approaches, [2,3], Concerningly, SMuRFless STEMI patients have an almost 50 % higher early mortality rate compared to patients with ≥1 SMuRF, a finding that is most pronounced in women [2]. Clearly, there is room for improvement in identifying this hidden, vulnerable group of patients in the primary prevention setting.

Large-scale genome-wide association studies (GWAS) have demonstrated that the majority of genetic loci associated with CAD are not associated with traditional risk factors and only ∼50 % of the identified loci have any predicted relationship with biological pathways known to be related to CAD [4]. The majority of identified loci are common variants with a minor allelic frequency of >5 %; although low-frequency variants with potentially greater impact have also been identified. Combined, these loci result in 30–40 % of the observed heritability of CAD [5]. The development of polygenic risk scores (PRS), which now incorporate hundreds of thousands of single-nucleotide polymorphisms (SNP), can predict CAD events largely independent of traditional risk factors/scores holds promise in terms of identifying people at high risk of CAD currently missed by traditional risk scoring [6,7], Many SNPs identified in CAD GWAS have low effect sizes; however, metanalysis derived scores that incorporate a larger number of SNPs, including those with low effect sizes, have improved the predictive performance PRS with fewer SNPs [8]. The lack of known relationship with traditional risk factors (blood pressure, lipid levels, diabetes and smoking, and family history) suggests PRS may have additional value in predicting risk, with the greatest impact potential in those considered low or intermediate risk based on traditional risk factor scores incorporating SMuRFs. Individuals with a top quintile PRS have an odds ratio of an acute coronary event (ACE) of 1.9 (95 % confidence interval [CI] 1.8 – 2.0) compared to individuals with a non-top quintile PRS [9,10], The relative risk reduction obtained with statin therapy in top quintile PRS individuals is 46–48 % compared with 13–29 % in non-top quintile PRS individuals, and the absolute risk reduction obtained is 2.75 fold higher in top quintile individuals [11,12].

In this study, we seek to model the hypothetical impact of systematically incorporating a CAD PRS into the primary prevention risk assessment workflow on, “ACE” cases and mortality, as well as the cost-effectiveness of this in a contemporary Australian healthcare context. We use a dynamic simulation model of CAD in the Australian population, calibrated to the experience for the ten years 2011–2020. Various scenarios and assumptions agreed upon by a panel of clinical and public health experts are used to estimate the impact that a CAD-PRS could have had if it had been available and implemented during those years.

## 2 Methods

### 2.1 Participatory model

**Figure 1:**
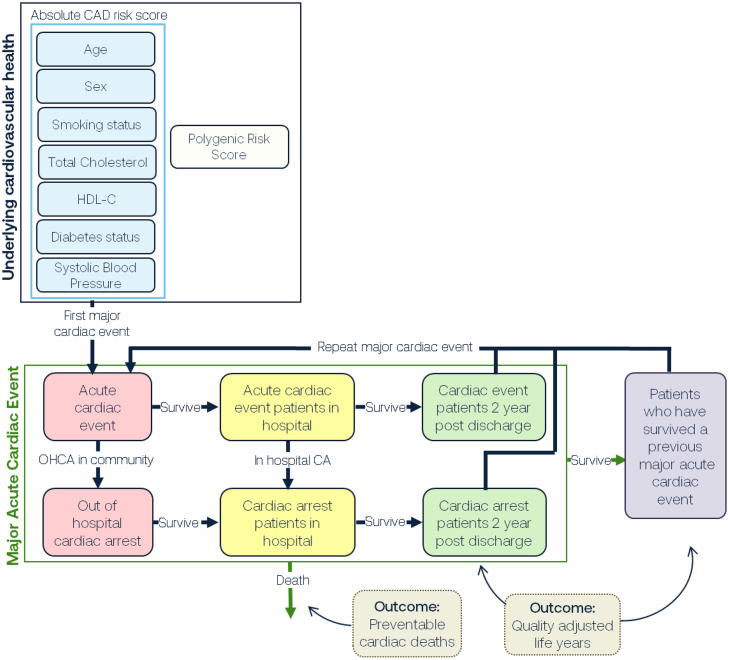
Simplified graphic representation of the model structure.

System dynamics (SD) modelling is founded on two key premises. Firstly, that systems are complex, meaning nonlinear relationships exist between components of the systems, including feedback loops and potentially long delays between cause and effect [13]. Secondly, SD modelling uses a participatory process to elicit qualitative understandings from key actors and quantifies these understandings with data and research evidence [14]. These principles allow SD modelling to provide accurate predictions of the effects of interventions within complex systems, such as healthcare. SD models have previously been used in a variety of scenarios in the CVD space, including clinical management, behavioural support, health access and regulations, and have been able to model outcomes of interest over time [15,16],

In this study we utilised an established whole-of-system model that was developed to inform public health strategies aimed at reducing the burden of CVD in Australia [17]. As previously described, the SD was developed in four stages: 1) participatory systems mapping and conceptual diagram development; 2) conversion of the conceptual diagram to a computational model via parameterisation with numeric inputs; 3) design, integration, and testing of strategies; and 4) model validation and uncertainty analysis. The multidisciplinary consortium developing this model included experts in public health research, clinical medicine, consumer groups, industry, health economics, and policy agency representatives. The model construction and analysis were performed using Stella Architect ver. 1.9.2 (www.iseesystems.com). A simplified graphical representation of the model is presented in Fig. 1. The population entered the models through ageing and migration and exited through deaths due to acute coronary event (ACE) and non-ACE reasons (to account for competing risks of deaths from non-ACE causes). We utilised real-world demographic and observed ACE data for Australians between 2011 and 2020 as the baseline scenario [18]. The model time horizon was 10 years from intervention implementation. Probabilities used in the models were derived from cohort studies and registries, and were calibrated against cause of deaths recorded on the Australian Bureau of Statistics (ABS) and primary diagnosis recorded on the National Hospital Morbidity Database (NHMD) [19].

The models divide the Australian population, aged 20 and older, into 10, 368 sub-populations stratified by: gender (2 levels); age (6 levels: 20–34, 35–44, 45–54, 55–64, 65–74 and 75+); total cholesterol (3 concentration levels and 2 treatment levels); high-density lipoprotein cholesterol (2 concentration levels), systolic blood pressure (4 levels and 2 treatment levels); diabetic status (3 levels: normal glucose regulation, impaired glucose tolerance, and type 2 diabetes mellitus); and smoking status (3 levels: never smoked, ex-smoker, current smoker). These data were obtained from ABS data, [18] the 2011 National Health Survey, [20] and the National Diabetes Services Scheme [21]. Within each of these 10, 368 risk cells, the 1-year probability of having an acute coronary event (ACE) is calculated using a method based on the Framingham 2008 ASCVD 10-year Risk Calculator [22].

At each timestep, a proportion of the people in each cell experiences an ACE. Some of these events are modelled as escalating to out-of-hospital cardiac arrests (OHCA), with incidence and initial survival probabilities based on data in the NSW Cardiac Arrest Registry reports [23]. The remainder are subject to a high 30-day mortality rate representing the risk of dying in hospital [24]. The next two years are treated as a high-risk period; the age-specific mortality rates are 1.425 times greater for men who did not have an OHCA, 1.495 times greater for women who did not have an OHCA, and 3.9 times greater for men and women that had an OHCA [[25], [26], [27]]. People also have a higher chance of experiencing a second ACE during this period, with a 25 % chance of a subsequent ACE event requiring hospital admission within two years of the initial event [27]. If they survive the two-year high risk period, people are moved to a population with previous CHD; this has a different set of ACE probabilities, based on another Framingham model, but is otherwise similar to the structure for people's first (‘index’) ACE described above [22]. Survivors of a subsequent ACE are recycled back into this sub-population (Fig. 1). A full description of the model calibration is provided in the Supplementary Material, ‘calibration of risk engine’.

Individuals with a high (defined as top quintile) PRS have an odds ratio of ACE of 1.9 (95 % confidence interval [CI] 1.8 – 2.0) compared to individuals with a non-top quintile PRS [7,9,10], We assessed the effect of varying the odds ratio of CAD in those with >80th percentile PRS vs < 20th percentile PRS within the reported 95 % CI [10]. Given the odds ratio of an ACE is 1.9 and that a 46–48 % relative risk reduction with statin treatment in top quintile PRS compared to non-top quintile PRS, we estimated that statin treatment in high PRS individuals would reduce their probability of an ACE to that of an non-top quintile PRS individual [7,11,12], The cost per annum for statin use was $203.92 based on the market share for the five most commonly prescribed statins in Australia and the average price of each of those statins using 2019–20 Prescriber Benefit Scheme data. The FRS 5-year risk was used as this is most consistent with the models currently used in primary prevention in the Australian healthcare setting.

### 2.2 Statistical analysis

We present a baseline integration, representing the actual experience over the ten years 2011–2020, and three scenarios for incorporation of the CAD PRS in primary prevention. The three scenarios are:

1. A ‘maximal’ implementation scenario, with everybody over the age of 35 years old having a PRS calculated, and all people with a high PRS (top quintile) are prescribed, and adhere to, a statin for primary prevention prophylaxis throughout the ten year period. This scenario estimates the maximum benefit that could be achieved through integrating the PRS into primary prevention in Australia in terms of ACEs prevented and lives saved assuming a constant preventive benefit from the statin in the PRS population.
2. A ‘targeted’ implementation scenario with pragmatic assumptions regarding the uptake of Heart Health Checks (HHC), the uptake of PRS, and prescription of statins in response to high PRS. Specifically, the targeted approach and assumptions included in this model are:
  a. Only people ≥45 years old are eligible for a HHC;
  b. Only some eligible people will have a HHC each year (5 % of 45–54 year olds, 20 % of ≥55 year olds);
  c. Only people aged 45–74 years old with a low to medium ‘traditional’ risk score (i.e., FRS 5-year risk ≤15 %) are eligible to have a PRS;
  d. 75 % of those eligible for a PRS will be referred for a PRS;
  e. 75 % of those found to have a high PRS will be prescribed a statin; and
  f. Graded statin adherence and discontinuation. Compliance/adherence rates were determined from the 2011 National Health Survey and varied according to age and sex (Supplementary Table 2).
3. An ‘intermediate’ implementation scenario, in which all eligible people have their PRS calculated and most have it acted upon. The specific assumptions in this model are:
  a. All people aged 45–75 years old attend a HHC and have their FRS calculated;
  b. All those with a low to medium ‘traditional’ risk score (i.e., FRS 5-year risk ≤15 %) have a PRS performed;
  c. 75 % of those found to have a high PRS are prescribed a statin; and
  d. Graded statin adherence and discontinuation (Supplementary Table 2).

### 2.3 Health economic measures assessed

The primary outcome was net cost per quality-adjusted life years (QALY) gained, using a standard conversion factor of AU$50, 000, a value broadly considered to be a threshold of willingness to pay for 1 QALY in the health sector [28]. Secondary outcomes assessing the economic impacts of the three approaches included: ACE-related healthcare costs over the ten years, including ambulance, hospital, pharmacy and rehabilitation costs; total cost of PRS tests performed (based on a US$100 unit cost [29], which translates to A$147.20 using a standard conversion factor); and net cost, which represents the excess in healthcare expenditure above the baseline ACE-related health costs. The delivery of PRS results is captured within the cost of the Heart Health Check, in line with Australian Medicare item numbers that state that the discussion of results of a test form part of the consultation fee when the decision is made to perform the test and/or the item number for the diagnostic test itself. Changes in management based on the test may result in additional consultations which are captured in the excess in healthcare expenditure above the baseline ACE-related health costs. Net costs include: the cost of running the PRS tests themselves, the costs of commencing additional people on statin therapy, and the cost savings due to fewer ACS events. Net benefit was defined as the value of QALYs saved minus the net cost.

### 2.4 Patient and public involvement

This study was derived as part of the Australia National Health and Medical Research Council Centre of Research Excellence for Coronary Artery disease. This program of work and this study was established with extensive patient and stakeholder involvement regarding individual and community healthcare priorities.

## 3 Results

Primary and secondary study outcomes are presented in Table 1. Baseline conditions reflect the historical data pattern of CVD deaths and hospitalisations in Australia between 2011 and 2020, without the incorporation of a CAD PRS in the primary prevention program. The total healthcare costs associated management of ACEs in Australia over the 10-year period was $19, 218 million. During this period, there were 541, 685 ACE cases, and 125, 683 ACE deaths. Unsurprisingly, the maximal scenario, in which everybody over the age of 35 years old have a PRS calculated and all people with a high PRS are prescribed and continue a statin for primary prevention prophylaxis as prescribed throughout the ten years, has the highest net cost ($3, 404 million) and also has the greatest number of ACE cases and deaths averted (60, 284 and 12, 374 respectively).

**Table 1:** System dynamics model outputs.

Measure	Baseline	Maximal	Intermediate	Targeted and Pragmatic
1 Health care costs $m	19,218	22,116 [22,001 – 22,235]	20,311 [20,271 – 20,352]	19,539 [19,521 – 19,556]
2 PRS cost $m	–	506	332	160
3 Net cost $m	–	3404 [3289 – 3523]	1425 [1385 – 1466]	480 [463 – 498]
4 QALYs	2616,392	183,682 [166,087 – 200,694]	63,400 [57,310 – 69,291]	24,085 [21,765 – 26,331]
5 Value of QALYs gained $m	–	9184 [8304 – 10,035]	3170 [2866 – 3465]	1204 [1088 – 1317]
6 Cost per QALY gained $	–	18,531 [16,388 – 21,210]	22,470 [19,983 – 25,579]	19,945 [17,592 – 22,885]
7 Net benefit $m	–	5780 [4782 – 6746]	1745 [1400 – 2080]	724 [590 – 853]
8 ACE deaths averted	125,683	12,374 [11,189 – 13,520]	3801 [3434 – 4155]	2052 [1854 - 2244]
9 ACE cases averted	541,685	60,284 [54,508 – 65,868]	20,850 [18,843 – 22,791]	9335 [8435 – 10,207]

[ ] represents when the odds ratio of CAD in those with > 80th percentile PRS vs < 20th percentile PRS is varied within the 95 % CI reported by Aragam et al. (2020).^10^.

**Figure 2:**
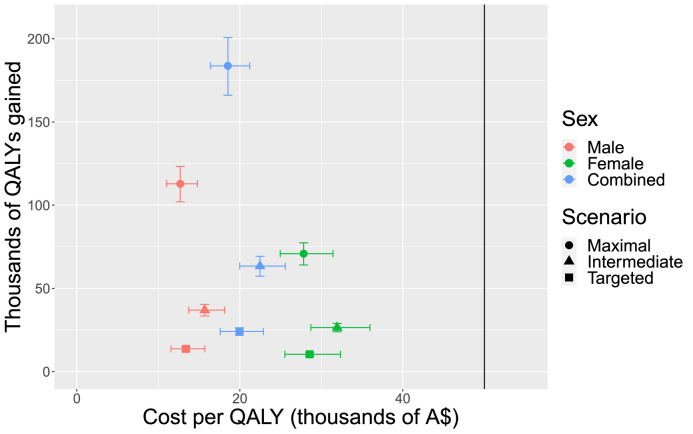
Costs per quality adjusted life year (QALY) vs thousands of QALYs gained. Dots represent maximal implementation scenario; triangles represent intermediate implantation scenario; squares represent targeted and pragmatic implementation scenario; red represents males only; green represents females only; and blue represents males and females combined. In all scenarios the cost per QALY is below the $50, 000 willingness to pay threshold indicated by the solid vertical line; this holds true when the odds ratio of CAD in those with >80^th^ percentile PRS vs < 20^th^ percentile PRS is varied within the 95% CI reported by Aragam et al. (2020), as shown by the cross bars [10].

The cost per QALY gained was substantially less than $50, 000 in each of the three PRS use scenarios assessed (maximal, intermediate, and targeted/pragmatic) (Fig. 2& Table 1). Interestingly, the lowest cost per QALY gained is seen in the maximal PRS use model ($18, 531 per QALY gained). Additionally, in all scenarios the cost per QALY remains below the $50, 000 willingness to pay threshold when the odds ratio of CAD in those with >80th percentile PRS vs < 20th percentile PRS is varied within the 95 % CI reported by Aragam et al. (2020) (Fig. 2& Table 1) [10]. Together, this data demonstrates the expected cost effectiveness of incorporating the PRS in primary prevention HHC and suggests that the broader the use of the PRS in individuals aged 45–75 years old, the greater the benefit and cost effectiveness of the strategy.

We next conducted subgroup analyses to assess the cost effectiveness of the strategies in females and males (Table 2) and in those with a low traditional risk score (Supplementary Table 3). These analyses demonstrated that whilst the three PRS incorporation scenarios were cost effective in both males and females (as defined by cost per QALY gained <$50, 000), the cost per QALY gained is substantially lower in males when compared to females in each of the three scenarios (Fig. 2& Table 2). Although the selective use of PRS in those having a HHC found to be at low risk using a traditional risk score (FRS 5-year risk ≤10 %) was cost effective (Supplementary Table 3), there were no significant cost savings when compared to the same approach in individuals with low to medium traditional risk score (FRS 5 year risk ≤15 %) and the net benefit was reduced (Table 1).

**Table 2:** System dynamics model outputs by gender.

Measure	Baseline		Maximal		Intermediate		Targeted and Pragmatic	
	Males	Females	Males	Females	Males	Females	Males	Females
1 Health care cost $m	11,605	7614	12,790 [12, 715–12, 867]	9326 [9286– 9368]	12,021 [11,996–12,047]	8290 [8275–8305]	11,713 [11,703–11,724]	7825 [7.,819–7832]
2 PRS cost $m	–	–	247	259	163	169	75	86
3 Net cost $m	–	–	1432 [1358–1510]	1971 [1931–2013]	579 [554–606]	845 [830–860]	183 [173–194]	297 [291–304]
4 QALYs gained	1536,717	1079,675	112,842 [102, 034–123, 293]	70,840 [64,053–77,401]	36,939 [33,388–40,374]	26,461 [23,922–28,917]	13,674 [12,355–14,951]	10,411 [9411–11,380]
5 Value of QALYs gained $m	–	–	5642 [5102–6165]	3542 [3203–3870]	1847 [1669–2019]	1323 [1196–1446]	684 [618–748]	521 [471–569]
6 Cost per QALY gained $	–	–	12,694 [11,011–14, 797]	27,830 [24,952–31,425]	15,687 [13,727–18,136]	31,939 [28,716–35,967]	13,385 [11,545–15,684]	28,560 [25,536–32,339]
7 Net benefit $m	–	–	4210 [3592–4807]	1571 [1190–1939]	1267 [1064–1464]	478 [336–615]	501 [424–575]	223 [166–278]
8 ACE deaths averted	77,857	47,826	7652 [6919–8360]	4722 [4270–5160]	2298 [2077–2513]	1502 [1358–1642]	1162 [1049–1271	890 [804–973]
9 ACE cases averted	351,889	189,796	39,158 [35,406–42,786]	21,125 [19,101–23,083]	13,080 [11,821–14,300]	7769 [7023–8492]	5615 [5073–6141]	3720 [3362–4066]

[ ] represents when the odds ratio of CAD in those with > 80th percentile PRS vs < 20th percentile PRS is varied within the 95 % CI reported by Aragam et al. (2020).^10^.

## 4 Discussion

Despite effective primary prevention programs incorporating traditional risk scores being implemented in recent decades, CAD remains the leading cause of death worldwide [1]. PRS for CAD are able to detect individuals at risk of ACE who are not identified using traditional multivariable risk scores in clinical use today [6]. Using SD models we demonstrate the impact of incorporating PRS in a contemporary primary prevention setting in Australia and found it to be a highly cost-effective tool under a number of different implementation scenarios. Firstly, we demonstrated that pragmatically incorporating a CAD PRS in the primary prevention setting in middle-aged individuals already attending a HHC (which unfortunately currently only includes 5–20 % of the eligible population/year), that are determined to be at low or moderate risk based on traditional risk score, would prevent over 2, 000 deaths over ten years, and result in 24, 085 QALYs gained at a cost of $19, 945 per QALY. Incorporating the PRS more liberally, if all individuals aged 45–75 years old had a HHC and those with low or moderate FRS had a PRS performed, and acted upon, this would prevent 3, 801 deaths and result in 63, 400 QALY gained at a cost of $22, 470 per QALY. Finally, in an aspirational scenario, if all Australian adults over the age of 35 years old had a PRS performed (irrespective of traditional risk score), and all those in the top PRS quintile were prescribed and continued taking a statin, over 60, 000 ACE cases could be prevented over the ten-year period, with 183, 682 QALYs gained at a cost of $18, 531 per QALY. These three scenarios clearly demonstrate the cost effectiveness of incorporating a PRS in the Australian CVD primary prevention setting and highlight that the broader the uptake of the strategy, the greater the impact and the more cost effective the approach. Additionally, it is worth considering that as PRS is genetically determined it influences lifetime risk of cardiovascular disease and is a one off test that can continue to be incorporated in an individual's cardiovascular risk assessments beyond the time period assessed in these analyses.

This is the first health economic analysis of CAD PRS implementation performed using systems dynamic modelling. In contrast to a prior study that assessed the cost effectiveness of implementing a PRS by applying Canadian health care costs to a simulation utilising the UK-Biobank dataset, our study elegantly demonstrated the potential impact and cost effectiveness of incorporating PRS CAD in the contemporary Australian setting utilising real world health care costs, compliance data, and outcome data [30]. Our results are complementary to the findings obtained using a Markov model utilising PRS as a risk enhancer factor in the pooled cohort equation in a United States setting, which was also shown to be cost effective [29]. Systems dynamic modelling captures and accounts for interrelationships and dynamic changes within complex systems, accounting for much granularity that is not captured in many health economic models. A strength of the SD approach used is that model structure and parameter values suggested by the expert modelling consortium were cross-validated with the best available data. The SD modelling used in these analyses is eminently transferable to accurately assess the potential cost effectiveness of implementing other novel biomarkers or risk factors/scores for CAD. Adopting this modelling approach will allow implementation clinical trials to focus on markers that not only have clinical utility but are also cost effective.

Despite these strengths, there are inherently some limitations to the approach. Intrinsically any model remains a simplification of the real world and despite the best efforts to incorporate accurate data/estimates, the quality and quantity of data are invariably imperfect. Additionally, the models did not have stratifications of social determinants of health, including socio-economic status, and Indigenous status, and thus effects of strategies in these subpopulations specifically are not captured. The current models did not account for implementation costs including clinician education, expansion of laboratory capabilities, and promotional material. Additionally, whilst the three scenarios simulated different uptake rates for attending a heart health clinic, performing a PRS and acting on a high PRS, these scenarios do not necessarily capture the full heterogeneity in terms of adoption, reach and acceptability of incorporating a PRS in a real-world primary prevention setting. Future iterations of the SD model can be developed as the new evidence emerges, for example the ESCELATE trial which is an implementation clinical trial incorporating a polygenic risk score-triaged coronary calcium score into primary prevention [31]. There is ongoing debate regarding how best to integrate CAD PRS in to primary prevention, some advocate for its widespread adoption early in adulthood and then ongoing integration within a risk score combining traditional risk factors and the PRS throughout adulthood. The addition of PRS to a traditional risk based score has been shown to improve prediction above traditional risk scores, however the effect of adopting a PRS in this manner throughout adulthood has not been assessed and the health economics of such an approach is therefore beyond the scope of the current study [32]. There is ongoing work regarding the performance of genomic scores being applied in populations that may differ from the population that they were developed in. This is an important technical consideration that is currently being addressed in cohorts around the world. Despite this, the broad concept that top quintile PRS individuals are at elevated risk cardiovascular disease that was modelled in this study stands.

## 5 Conclusion

The incorporation of a CAD PRS in contemporary primary prevention programmes in Australia, such as in the Heart Health Check, would result in substantial health and societal benefits, and is cost effective. This study highlights that the broader the uptake of the CAD PRS in the primary prevention setting in middle-aged Australians, the greater the impact and the more cost effective the strategy.

## Sources of funding

NSW Health, NSW Cardiovascular Research Capacity Program, Senior Researcher Grant; New Frontiers in Personalised prevention of CAD (RFRHPI000110), Medical Research Future Fund – Frontier Health and Medical Research Initiative; National Health and Medical Research Council, Australia Partnership for Precision Prevention in CAD (GNT2005791); National Health and Medical Research Council, Australia Centre for Research Excellence- Better Outcomes in Coronary Artery Disease (APP1196629); National Health and Medical Research Council (NHMRC)- Practitioner Fellowship: From fundamental mechanisms to strategic methods of early detection and treatment for cardiovascular disease (GNT1135920); Discovering and translating new markers and mechanisms of atherosclerosis (NSW Health Cardiovascular Disease Clinician Scientist Grant), NSW Office of Health and Medical Research; Strategies for prevention of coronary artery disease, particularly in people without traditional risk factors, Heart Foundation Post-Doctoral Fellowship (Award ID: 106742).

## Disclosures

GF reports personal consulting fees from CSL and Janssen, and grants from Abbott Diagnostic outside the submitted work. In addition, GF has a patent Biomarkers and Oxidative Stress awarded USA May 2017 (US9638699B2) issued to Northern Sydney Local Health District. ZA disclose no conflict of interest. Dr Mujwara and Dr Bottà are employees of Allelica, Inc.

**Figure:**
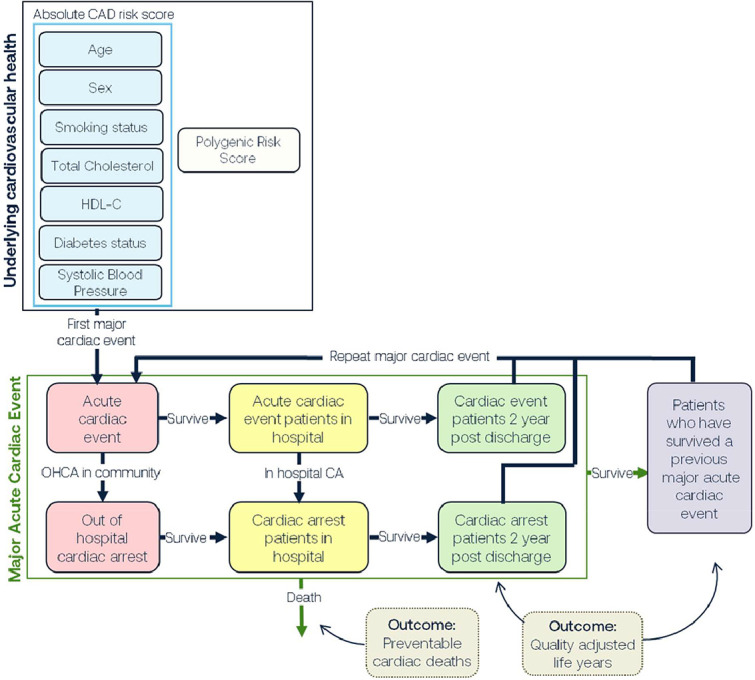
**Central Illustration:** Simplified representation of systems dynamic model inputs and outcomes.

Alt-text: Unlabelled box

## CRediT authorship contribution statement

**Stephen T. Vernon:** Writing – original draft, Methodology, Conceptualization. **Stuart Brentnall:** Visualization, Software, Methodology, Formal analysis, Data curation. **Danielle J Currie:** Writing – review & editing, Resources, Project administration, Methodology. **Cindy Peng:** Writing – review & editing, Resources, Project administration, Methodology. **Michael P. Gray:** Writing – review & editing, Project administration. **Giordano Botta:** Writing – review & editing, Investigation. **Deo Mujwara:** Writing – review & editing, Investigation. **Stephen J. Nicholls:** Writing – review & editing, Investigation. **Stuart M. Grieve:** Writing – review & editing, Investigation. **Julie Redfern:** Writing – review & editing, Investigation. **Clara Chow:** Writing – review & editing, Investigation. **Jean-Frederic Levesque:** Writing – review & editing, Methodology, Investigation. **Peter J. Meikle:** Writing – review & editing, Investigation. **Garry Jennings:** Writing – review & editing, Investigation. **Zanfina Ademi:** Writing – review & editing, Methodology, Investigation, Conceptualization. **Andrew Wilson:** Writing – review & editing, Supervision, Methodology, Investigation, Conceptualization. **Gemma A. Figtree:** Writing – original draft, Supervision, Methodology, Conceptualization.

## Declaration of competing interest

The authors declare the following financial interests/personal relationships which may be considered as potential competing interests:

Stephen Vernon reports financial support was provided by National Heart Foundation of Australia. If there are other authors, they declare that they have no known competing financial interests or personal relationships that could have appeared to influence the work reported in this paper.

## Appendix Supplementary materials

Image, application 1
