# Supplementary material for: Health economic analysis of polygenic risk score use in primary prevention of coronary artery disease – A system dynamics model
Source: Am J Prev Cardiol. 2024 May 18;18:100672. doi: 10.1016/j.ajpc.2024.100672 (PMC11143886; doi:10.1016/j.ajpc.2024.100672)
Supplement: Supplementary file 1 [file mmc1.docx]

# Supplementary Material:

***Calibration of risk engine***

# The 1-year probability of ACE for each risk cell was calibrated to the population-wide crude rates reported by the AIHW^[[1]](#footnote-1)^. For the sub-population who had not previously had an ACE, the underlying model is the Framingham model for the 10-year risk of CVD^[[2]](#footnote-2)^; we assume that the relative risks of ACE or CHD between risk cells are similar to those of CVD. This model is an instance of the Cox proportional hazards model, one of the assumptions of which is that these hazards are the same for all time horizons, so that we are justified in using the 10-year model for 1-year risks provided that it is correctly fitted.

# Specifically, the probability of a person having an event within $T$ years is modelled as

#

$$P_{0}\left( T \right)=1-S_{0}\left( T \right)^{\exp\left( L_{C}\left( \boldsymbol{x} \right) \right)},$$

# where $\mathbf{x}$ is a vector of the relevant risk factors for the individual, and the ‘linear predictor’ $L_{C}\left( \mathbf{x} \right)$ the linear combination of them defined by the ‘$\beta$’ coefficients that can be found on the Framingham web page. There are separate models for males and females.

# The ‘survival probability’ $S_{0}\left( T \right)$ is the probability of not having an event within $T$ years for a reference person for whom $L_{C}\left( \mathbf{x} \right)$ is zero; it is this parameter that we need to find in order to calibrate the model. We can do this if we know:

1. The total number of events $E_{0}$ in the population of interest over a given $T$-year period.
   1. In our case, with $T=1$, this is the number in 2011, the year at the start of the modelled period, which was derived from the AIHW numbers (though this is complicated by needing to take account of cases in people who *had* previously had an ACE, as discussed below).
2. The number of people $n_{k}$ in each risk cell $k$, in the population of interest. The index $k$ goes from 1 to 5,184 for males, and likewise for females, to give the total of 10,368 sub-populations.

If each person in cell $k$ is assigned the same vector $\mathbf{x}_{k}$ of risk factors, the parameter $S_{0}\left( T \right)$ can then be found by solving the equation

$$E_{0}=\sum_{k} n_{k}P_{0,k}\left( T \right)=N_{0}-\sum_{k} n_{k}S_{0}\left( T \right)^{\exp\left( L_{C}\left( \mathbf{x}_{k} \right) \right)},$$

where $N_{0}=\sum n_{k}$ is the total population (i.e., the number of men or women in the age range of interest who have not previously had an ACE). We found solutions numerically, using the base R optim() function^[[3]](#footnote-3)^. For an arbitrary reference person, a non-smoking non-diabetic of age 50 with total cholesterol 5.2 mmol L${}^{-1}$, HDLC 1.0 mmol L${}^{-1}$ and systolic blood pressure 130 mm Hg (and who is not on blood pressure medication), the one-year ACE probabilities $1-S_{0}\left( 1 \right)$ for 2011 were calculated to be 0.31% for males and 0.19% for females.

For the population who *have* had an ACE, we used the Framingham model for the 2-year risk of recurrent CHD^[[4]](#footnote-4)^. This is an instance of the Weibull accelerated life model, based on the Gumbel-maximum extreme value distribution^[[5]](#footnote-5)^, for which the $T$-year probability of an event is

$$P_{1}\left( T \right)=1-\exp\left[ -T^{p}\exp\left\{ -pL_{W}\left( \mathbf{x} \right) \right\} \right],$$

where the parameter $p$ to be fitted is known as the (reciprocal of the) ‘extreme value parameter’. The vector $\mathbf{x}$ of risk factors is slightly different from the one used for the above Cox model and contains fewer risk factors (so that the risk measure is less fine-grained); but the linear predictor $L_{W}\left( \mathbf{x} \right)$ is again a linear combination of these risk factors. Again, this is intended to be valid for any time horizon $T$, and we use the value $T=1$ year. The parameter $p$ isn’t as easy to interpret as $S_{0}\left( T \right)$, but can be found in a similar manner; we obtained the values 1.37 for males, and 1.18 for females.

For 2011, the crude rates for males and females aged 25 and older were 593 and 337 per 100,000 population, respectively. Taking account of the (near-)zero incidence in the 20-24 age group, these become 533 and 306 respectively for the 20 and older age group. These can easily be scaled up to total numbers using ABS population data, but in order to obtain values for $E_{0}$ and $E_{1}$ (the total number of events for the sub-population who *have* previously experienced an ACE) to use in the above calculations, we still need to apportion them between people who have or have not had a prior ACE. Little information seems to be available concerning the difference in ACE incidence in Australia between these two groups, but we have been able to make an estimate, as follows.

For men and women separately, we require two conditional probabilities: the probability $\mathbb{P}\left( \text{ACE} \right| \text{no prior CHD})$ of experiencing an ACE given that you have not previously done so (or developed CHD); and the corresponding probability $\mathbb{P}\left( \text{ACE} \right| \text{prior CHD})$ given that you *have*.

We are not aware of any data giving these values explicitly, but have found values for the related probability $\mathbb{P}\left( \text{prior CHD} \right| \text{ACE})$.^[[6]](#footnote-6)^ Bayes’ theorem then gives the required probabilities as:

$$\mathbb{P}\left( \text{ACE} | \text{prior CHD} \right)=\frac{\mathbb{P}\left( \text{prior CHD | ACE} \right)\mathbb{P}\left( \text{ACE} \right)}{\mathbb{P}\left( \text{prior CHD} \right)}$$

and

$$\mathbb{P}\left( \text{ACE | no prior CHD} \right)=\frac{\left[ 1-\mathbb{P}\left( \text{prior CHD | ACE} \right) \right]\mathbb{P}\left( \text{ACE} \right)}{1-\mathbb{P}\left( \text{prior CHD} \right)}.$$

The $\mathbb{P}\left( \text{ACE} \right)$ values are essentially the 533 and 306 given above, redistributed over the age groups assuming a polynomial (cubic) dependence on age. $\mathbb{P}\left( \text{prior CHD} \right)$ was based on age- and sex-specific data from Banks *et al.* (2016)^[[7]](#footnote-7)^. The results, all per 100,000, are summarised in the following table:

|  | **2011 ACE incidence, ages 20**$\boldsymbol{+}$ | |
| --- | --- | --- |
|  | **Men** | **Women** |
| $\mathbb{P}\left( \text{ACE} \right)$ | 533 | 306 |
| $\mathbb{P}\left( \text{ACE \vert no prior CHD} \right)$ | 443 | 253 |
| $\mathbb{P}\left( \text{ACE \vert prior CHD} \right)$ | 1,704 | 1,118 |

That gives us $E_{0}$ and $E_{1}$. The other ingredient in the calibration of the risk engine is the number $n_{k}$ of people in each risk cell $k$, again split into those with and without prior ACE; these numbers were also required for the initialisation of the SD model.

In the absence of more detailed data, we calculated the *total* in each cell, and divided it into the two subgroups using the $\mathbb{P}\left( \text{prior CHD} \right)$ values mentioned above. The totals were obtained from a variety of sources:

- Population data from the ABS web site^[[8]](#footnote-8)^
- Cholesterol data from the 2011 National Health Survey^[[9]](#footnote-9)^
  - Data was adequate to estimate the *joint* distribution of total cholesterol and HDLC, the two cholesterol values used in the Framingham model
  - It also had information about the number of people in each cell who were on lipid-modifying therapy, which was required for SD model initialisation but not for the risk engine itself
- Data on systolic blood pressure, and whether people were taking medication for hypertension, again from the 2011 NHS dataset
  - Again, it was possible to obtain estimates for the *joint* proportions
- Data on the proportion of Type 2 diabetics in each age/sex category, from the National Diabetes Services Scheme snapshot files^[[10]](#footnote-10)^
- Data on smoker status from the 2010 National Drug Strategy Household Survey^[[11]](#footnote-11)^

Except where noted, the proportions for each risk factor were multiplied together to give the proportions in each risk cell, that is, in lieu of any more detailed data, the risks were taken to be independent. This is a limitation that can be overcome if or when more detailed data become available.

***Calibration and validation of SD model***

The evolution over time of the population’s distribution of the risk factors is modelled using a standard system dynamics stock and flow methodology. There are separate sub-modules (sectors) for each of the five risk factors: smoking, diabetes, total cholesterol, HDLC and blood pressure. Within each sector there are compartments (stocks) for each level of the risk factor (see Supplementary Table 1).

For the cholesterol and blood pressure sectors, there are two stocks for each level, representing people who are or are not taking medication for the factor in question. For blood pressure, this is required because medication status is one of the predictors in the Framingham model. For cholesterol, it is required because commencing medication is the only response to a high PRS finding that we are considering. The impact of initiating statins is modelled by transferring a certain proportion of people from an untreated stock to the treated stock at the next lower cholesterol risk level.

The time evolution is affected by specifying the fraction of each stock that flows to each accessible stock per unit time. The fractions were initially specified using expert opinion and values from the literature, but were then modified using tuning parameters to calibrate more accurately to observations of the stock sizes for the period 2011-2020. Where possible, these observations were taken from the same sources as were used for the $n_{k}$ values described above, most of which have data for more than one year.

The calibration process consists of adjusting the tuning parameters by re-running the model until the target time trajectory for the stock is achieved to within acceptable accuracy, as determined visually by plotting graphs of the observed and modelled stocks (including uncertainty intervals where available). This process is repeated for each age/sex category within each stock.

Separate calibrations of $S_{0}$ and $p$ were not done for each year, partly because the required data are not available for all years, but the incidence of ACE continued to decline rapidly in the period covered. For males, the incidence for ages 20 and above calculated from the AIHW time series declined from 533 per 100,000 in 2011 to 416 in 2018, while the incidence for females declined from 306 to 212. This reduction is not likely entirely to be due to changes in the risk factors represented by the model; for instance, Mannsverk et al. (2016)^[[12]](#footnote-12)^ estimated that only 66% of the reduction in the incidence of acute CHD observed in their study population could be ascribed to trends in modifiable risk factors, the other 34% being due to other reasons. Hence, even if the SD model was modelling the risk factor distributions exactly, the calculated ACE rates would not track the observed ones exactly. For these reasons, the ACE rates returned by the risk module are multiplied by a factor which (in the baseline integration) brings them back into line with the AIHW rates, before being used in the top-level model. The key features of the model – the representation of the relative risks within the population, and of the change in the risk distribution resulting from the proposed introduction of PRS – are not affected by this adjustment.

**Supplementary Table 1:** Representative values used for each cell of the continuous factors in risk calculation

| **Risk factor** | **Risk level** | **Range** | **Representative value** |
| --- | --- | --- | --- |
| **Total cholesterol**  **mmol / litre** | Low | < 4 | 3.8 |
|  | Medium | 4 – 7.0 | 5.2 |
|  | High | > 7.0 | 7.1 |
| **HDL-C**  **mmol / litre** | Lower | $\geq$ 1 | 1.25 |
|  | Higher | < 1 | 0.75 |
| **Systolic BP**  **mm Hg** | Low | < 120 | 110 |
|  | Low-medium | 120 – 139 | 129.5 |
|  | Medium-high | 140 – 179 | 159.5 |
|  | High | $\geq$ 180 | 190 |
| **Diabetes** | Low – normal glucose regulation | | Both coded as non-diabetic for Framingham |
|  | Medium – impaired tolerance (‘pre-diabetic’) | |  |
|  | High – Type 2 diabetes | | |
| **Smoking** | Low | Never smoked | Both coded as non-smokers for Framingham |
|  | Medium | Ex-smoker |  |
|  | High | Current smoker | |

**Supplementary Table 2:** Annual statin “quit rate” as determined by the 2011 National Health Survey

|  | **20-34** | **35-44** | **45-54** | **55-64** | **65-74** | **75+** |
| --- | --- | --- | --- | --- | --- | --- |
| **Males** | 0 | 83% | 83% | 83% | 42% | 42% |
| **Females** | 42% | 42% | 42% | 42% | 42% | 21% |

**Supplementary Table 3:** System dynamics model outputs limited to low traditional risk individuals.

| **Measure** | **Baseline** | **Intermediate** | **Targeted and Pragmatic** |
| --- | --- | --- | --- |
| 1 Health care cost $m | 19,218 | 20,425 | 19,560 |
| 2 PRS cost $m | - | 332 | 140 |
| 3 Net cost $m | - | 1,539 | 482 |
| 4 QALYs gained | 2,616,392 | 48,568 | 14,353 |
| 5 Value of QALYs gained $m | - | 2,428 | 718 |
| 6 Cost per QALY gained $ | - | 31,694 | 33,594 |
| 7 Net benefit $m | - | 889 | 235 |
| 8 ACE deaths averted | 125,683 | 2,499 | 973 |
| 9 ACE cases averted | 541,685 | 14,897 | 4,939 |

1. Table **CHD**, cells F86:G103, in **AIHW-CVD-92-Heart-stroke-and-vascular-disease-Australian-facts-data-tables.xlsx**, available at <https://www.aihw.gov.au/reports/heart-stroke-vascular-diseases/hsvd-facts/data> [↑](#footnote-ref-1)
2. <https://framinghamheartstudy.org/fhs-risk-functions/cardiovascular-disease-10-year-risk/> [↑](#footnote-ref-2)
3. R Core Team (2021). R: A language and environment for statistical computing. R Foundation for Statistical Computing, Vienna, Austria. URL <https://www.R-project.org/> [↑](#footnote-ref-3)
4. <https://framinghamheartstudy.org/fhs-risk-functions/recurrent-coronary-heart-disease/> [↑](#footnote-ref-4)
5. Inferred from D'Agostino, Ralph B., et al. "Primary and subsequent coronary risk appraisal: new results from the Framingham study." American heart journal 139.2 (2000): 272-281. [↑](#footnote-ref-5)
6. See cells B20, E20, B40 and E40 of the Table B2 tab in the spreadsheet that can be found <https://www.aihw.gov.au/getmedia/acbf0446-05c9-4693-b834-98877dba4ac9/aihw-cdk-9.pdf.aspx?inline=true>- this is for 25-84 year olds in New South Wales and Victoria in 2012-2015, so we need to assume that the value doesn’t vary much across time, location or age. The values are $5,817/25,429=0.229$ for males, and $2,645/11,917=0.222$ for females. [↑](#footnote-ref-6)
7. Banks, Emily, et al. "Absolute risk of cardiovascular disease events, and blood pressure‐and lipid‐lowering therapy in Australia." Medical Journal of Australia 204.8 (2016): 320-320. [↑](#footnote-ref-7)
8. <https://www.abs.gov.au/statistics/people/population/national-state-and-territory-population/sep-2020#data-download> [↑](#footnote-ref-8)
9. <https://www.abs.gov.au/AUSSTATS/abs@.nsf/Lookup/4324.0.55.001Main+Features492011-12?OpenDocument> [↑](#footnote-ref-9)
10. <https://www.ndss.com.au/about-the-ndss/diabetes-facts-and-figures/diabetes-data-snapshots/> [↑](#footnote-ref-10)
11. <https://www.aihw.gov.au/about-our-data/our-data-collections/national-drug-strategy-household-survey/2010-national-drug-strategy-household-survey> [↑](#footnote-ref-11)
12. Mannsverk, Jan, et al. "Trends in modifiable risk factors are associated with declining incidence of hospitalized and nonhospitalized acute coronary heart disease in a population." *Circulation* 133.1 (2016): 74-81. [↑](#footnote-ref-12)
